# Native and foreign born as predictors of pediatric asthma in an Asian immigrant population: a cross sectional survey

**DOI:** 10.1186/1476-069X-6-13

**Published:** 2007-05-02

**Authors:** Doug Brugge, Angela C Lee, Mark Woodin, Christine Rioux

**Affiliations:** 1 Department of Public Health and Family Medicine, Tufts University School of Medicine, Boston, MA, USA; 2 Jonathan M. Tisch College of Citizenship and Public Service, Tufts University, Medford, MA, USA; 3 Department of Civil and Environmental Engineering, School of Engineering, Tufts University, Medford, MA, USA

## Abstract

**Background:**

Asthma prevalence is lower in less developed countries and among some recent immigrant populations in the US, but the reasons for this are not clear. One possibility is that early childhood infections are protective against asthma.

**Methods:**

We surveyed Asian immigrant children (n = 204; age 4–18) to assess the relationship between asthma and native or foreign place of birth. We included questions about environmental exposures, demographic variables and family history of asthma to test whether they might explain effects of place of birth on asthma.

**Results:**

The native and foreign born groups were similar in most respects. Analysis of association with diagnosed asthma for all ages together resulted in two logistic regression models. Both retained born in the US (ORs were 3.2 and 4.3; p < 0.01) and family history of asthma (ORs were 6.4 and 7.2; p < 0.001). One model retained living near heavy motor traffic (OR = 2.6; p = 0.012). The other retained language (OR = 3.2; p = 0.003). However, for older children (11–18 years of age) being born in the US lost some of its predictive power.

**Conclusion:**

Our findings are consistent with early childhood infections that are prevalent outside the US protecting against asthma.

## Background

Asthma continues to be a highly prevalent illness in the US with substantial personal and societal impact [1]. Understanding of the pathogenesis of asthma, however, remains uncertain and there is evidence that some subpopulations appear to be at greater risk than others. It is generally accepted that asthma etiology involves a complex matrix of genetic, immunological, socioeconomic, and environmental factors. Environment can be broadly defined to mean exposure to pollution, bacterial, viral, and parasitic infections, allergens such as cockroach or dust mite, family or household conditions such as number of siblings, attendance at day care, or growing up on a farm all of which are factors that have been associated with higher or lower incidence of asthma [2,3].

The relative contribution of genetic factors to asthma has been estimated to be from 40%–60% [4]. While mutations in a number of genes have been studied for associations with asthma, only a limited number have shown associations in a consistently reproducible manner. A number of theories have been proposed to explain the increased incidence of asthma in recent decades as well as differences in prevalence between populations. These include air pollution exposure [5-7]; decreases in exercise and increases in obesity [8,9]; and changes in early childhood infections [10,11]. The "Hygiene Hypothesis" is the theory that the increase in asthma and other allergic diseases in developed countries is explained by a decrease in infections, particularly childhood infections [12,13,8]. A strength of this hypothesis is that it is consistent with lower asthma prevalence in less developed countries and among some recent immigrant populations in the US [14] and in Australia [15].

The hygiene hypothesis has also been proposed to explain the higher prevalence of autoimmune and allergic diseases in more affluent western and industrialized countries. Recent studies have reported a potential molecular basis for the hygiene hypothesis noting a mutation in an atopy susceptibility gene, *Havcr2*, which may explain the inverse relationship between hepatitis A infection and the development of asthma [16-18].

Immigrant populations are potentially interesting populations in which to explore differences in asthma prevalence. US Asian populations have only recently begun to be examined in terms of asthma prevalence [19,20]. We have previously reported asthma prevalence in a predominantly Chinese immigrant population, observing that Asian children had lower risk than non-Asian children [21]. Subsequently, we showed that linguistic issues could affect reported prevalence of wheeze, indicating that estimates of asthma prevalence across language groups may be problematic [22,23]. We also observed that asthma prevalence was substantially higher among US born Chinese children than among those who were foreign-born [22].

This study builds on our prior work by verifying the findings and, more importantly, assessing the role of multiple environmental factors not considered in the earlier study, including: tobacco smoke, household pests, living near heavy traffic and family history of asthma.

## Methods

### Procedures

The Tufts-New England Medical Center Institutional Review Board and the South Cove Community Health Center Board of Directors approved the study protocol, which was considered minimal risk. Consent was given verbally at time of entry from parents/grandparents/guardians of children less than 11 years of age and the child him/herself for years thereafter. Families were provided with a written description of the study in their choice of English or Cantonese. Data collected were anonymous and de-identified for ease of data collection while complying with HIPAA regulations which precluded collection of town or neighborhood of residence.

### Survey instrument

A written questionnaire was administered to all participants in the study as a tool for assessing asthma status. Parents/grandparents/guardians served as proxies for children younger than 11 years of age. Children ages 11–18 completed their own questionnaire. Our reasoning was that young children would more likely be unable to answer accurately and that parents would be less knowledgeable of the health of their older children. The survey was written in English and translated into Chinese by one translator using traditional Chinese characters. A second translator then translated back into English to ensure accuracy. Staff bilingual in Cantonese and English reviewed the original English questionnaire, the Cantonese translation and the second English translation to finalize both versions of the survey instrument.

Respondents chose to take the survey in either Cantonese or English. A bilingual English/Cantonese speaker (author ACL) verbally administered most of the questionnaires and answered questions when prompted by participants. An English speaker verbally administered a fraction (n = 71) of the questionnaires in English. Both surveyors received the same training and instructions, both were undergraduate students, both were Asian American and both were female. The questionnaire consisted of 26 questions.

Basic demographics were determined. Questions were asked regarding the child's sex, age, race, place of birth and age of immigration to the United States if born outside the US. Preferred language was inferred from choice of Chinese or English surveys. For children under 11, the preferred language reflects the choice of his/her parents. For children ages 11 and older, the language is the child's preferred language.

Information was collected on risk factors for asthma and on environmental factors. Respondents were asked "Does anyone in your household smoke?"; "Did you/the child's mother smoke while pregnant with the child?"; "Do you live near heavy motor traffic?"; "Are there visible indications of cockroaches, mice or rats in or around your home?"

The only primary outcome variable for the regression and tabular statistical analyses was diagnosed asthma, as determined from the validated Brief Pediatric Asthma Screen questionnaire [24]. Our prior work on translating asthma terms into Cantonese suggests that there are multiple translations of the word "wheeze", a term used is assessing possible undiagnosed asthma [22]. Therefore, we offered respondents multiple translations.

We tested the internal consistency of our primary outcome variable, diagnosed asthma, against key asthma symptoms on our questionnaire. The association of wheeze with diagnosed asthma had an odds ratio of9.0 (p < 0.001). For dry cough versus diagnosed asthma the odds ratio was 5.1 (p < 0.001). We interpret these as indicators that our translation of the diagnosed asthma question was effective, well understood and measured asthma.

We also asked, "Does any of your/your child's immediate family (mother, father, sister, brother) have asthma?" In addition, we asked "Have you/your child ever been diagnosed with allergies?"

### Sample

Of the eligible patients approached during recruitment, 12 declined to participate in the questionnaire for a response rate of 97%. A sample of 410 children ages 0–18 was recruited in the waiting rooms of the pediatrics departments of two health centers located in Boston Chinatown, Massachusetts from June 22 – July 25, 2005. We enrolled 187 children at South Cove Community Health Center, which specializes in care for non-English speaking low-income Asian immigrants. The remaining 223 children were enrolled at Tufts-New England Medical Center, which has an Asian clinic, but also treats a more general urban population. Patients who spoke neither English nor Cantonese or who had previously taken the screening test were ineligible.

For this analysis, we discarded questionnaires filled out by non-Asian respondents (n = 159) and those for children younger than 4 (n = 42). Children younger than 4 years of age were excluded because asthma diagnosis is uncertain at very young ages. Of the remaining 209 surveys, we discarded an additional 5 that had 3 or more missing answers. Our final database for the analysis reported here consisted of 204 Asian children, ages 4–18.

### Socioeconomic status classification

The occupations of both parents were recorded at the time of administering the questionnaire as a proxy for SES because our previous experience had taught us that child respondents poorly recalled education level of parents and key informants felt that we would not get accurate information on income in this population. Each occupation was classified as either "low SES" or "high SES". The classification of most jobs was clearly one category or the other. For example, jobs involving manual labor or requiring little education were categorized as low SES, whereas office-type jobs requiring more education were classified as high SES. Examples of low SES occupations included restaurant wait staff or factory workers. Examples of high SES occupations included teacher or engineer.

However, there were a handful of occupations that were difficult to classify. Examples included a Pilates instructor and a bodyguard. In order to produce a dichotomous variable, families that had one parent categorized as low SES and one parent categorized as high SES were assigned to the high SES category. Due to lack of recall by some children of their parents' occupations or the unemployed status of parents, we lacked occupational information on some respondents. We could assign an SES to 192 of the 204 respondents (6% missing), an improvement over the 25% missing data we obtained in a previous survey that depended on parental education [22].

### Statistical analysis

Data were double entered into SPSS (v12.0, SPSS Inc., Chicago, IL). Non-matching entries were identified as mistakes and then corrected using the original hardcopy version of the questionnaire. All variables were examined with standard descriptive statistics. Where appropriate, normality was assessed and outlying values analyzed. In general, no major outliers or significant departures from normality were detected and analysis proceeded to tabular and predictive models. The outcome variable of interest was "diagnosed with asthma", which was either yes or no. A variety of predictor variables were examined for association with the outcome variable using 2 × 2 tabular analysis and univariate logistic regression. Variables that showed no significant impact on asthma diagnosis in either tabular analysis or when analyzed in univariate logistic regression models were dropped from future analyses. Finally, a multivariable logistic regression model was built using variables remaining from the tabular and univariate analyses. The multivariable model was constructed by entering variables one at a time and watching for confounding or multicollinearity effects (a situationwhere more than one variable in a model is describing essentially the same linear effect).

## Results

### Descriptive values

Demographic categories and environmental and familial factors for foreign and US born and for the total sample are presented in Table 1. A higher percentage of US born children were from one of the clinical sites, however the difference did not reach statistical significance (p = 0.08). Respondents for US born children (children themselves over 11 and parents of children under 11) were slightly less likely to take the survey in Chinese, but again the difference was not statistically significant (p = 0.19). Almost half of surveys were for children under 11 in both native and foreign-born groups. There was a slightly higher percentage of females in the US born group, but the difference was not statistically significant (p = 0.13). High SES was more prevalent for the US born group (18.5% vs. 8.1%; p = 0.06).

**Table 1:** Descriptive values for foreign and US born members of the study population

	**Foreign Born**	**Born in the USA**	**P value**	**Total (n = 204)**
**Site**			*0.084*	
South Cove	80.0% (n = 52)	68.3% (n = 95)		72.1% (n = 147)
New England Medical Ctr.	20.0% (n = 13)	31.7% (n = 44)		27.9% (n = 57)
**Language of Respondent**			*0.191*	
Chinese	49.2% (n = 32)	59.0% (n = 82)		55.9% (n = 114)
English	50.8% (n = 33)	41.0% (n = 57)		44.1% (n = 90)
**Race of Child**				
Asian	100.0% (n = 65)	100.0% (n = 139)		100.0% (n = 204)
**Age of Respondent**			*0.374*	
Answered by parent/guardian	41.5% (n = 27)	48.2% (n = 67)		46.1% (n = 94)
Answered by child > 11	58.5% (n = 38)	51.8% (n = 72)		53.9% (n = 110)
**Sex of Child**			*0.130*	
Male	52.3% (n = 34)	41.0% (n = 57)		44.6% (n = 91)
Female	47.7% (n = 31)	59.0% (n = 82)		55.4% (n = 113)
**Child with Diagnosed Asthma**			*< 0.001*	
No	90.8% (n = 59)	66.9% (n = 91)		74.6% (n = 150)
Yes	9.2% (n = 6)	33.1% (n = 45)		25.4% (n = 51)
**Child with possible undiag. Asthma**			*0.476*	
No	93.8% (n = 61)	96.3% (n = 130)		95.5% (n = 191)
Yes	6.2% (n = 4)	3.7% (n = 5)		4.5% (n = 9)
**Child with diag. Allergies**			*0.183*	
No	79.7% (n = 51)	70.8% (n = 97)		73.6% (n = 148)
Yes	20.3% (n = 13)	29.2% (n = 40)		26.4% (n = 53)
**Child with family members who have asthma**			*0.007*	
No	96.8% (n = 61)	83.2% (n = 114)		87.5% (n = 175)
Yes	3.2% (n = 2)	16.8% (n = 23)		12.5% (n = 25)
**Home smoking**			*0.697*	
No	55.4% (n = 36)	58.3% (n = 81)		57.4% (n = 117)
Yes	44.6% (n = 29)	41.7% (n = 58)		42.6% (n = 87)
**Mother smoked during pregnancy**			*0.328*	
No	100.0% (n = 64)	96.4% (n = 134)		97.5% (n = 198)
Yes	0.0% (n = 0)	3.6% (n = 5)		2.5% (n = 5)
**Pests seen in or around the home**			0.292	
No	64.6% (n = 42)	56.8% (n = 79)		59.3% (n = 121)
Yes	35.4% (n = 23)	43.2% (n = 60)		40.7% (n = 83)
**Home near heavy motor traffic**			*0.283*	
No	56.9% (n = 37)	64.7% (n = 90)		62.3% (n = 127)
Yes	43.1% (n = 28)	35.3% (n = 49)		37.7% (n = 77)
**Family SES**			*0.060*	
Low SES	91.9% (n = 57)	81.5% (n = 106)		84.9% (n = 163)
High SES	8.1% (n = 5)	18.5% (n = 24)		15.1% (n = 29)

Asthma prevalence was substantially higher in the US born group (33.1% vs. 9.2%; p < 0.001), consistent with our survey a year earlier in one of the two clinics in this study [22]. Possible undiagnosed asthma was found in a higher percentage of the foreign born children, but the difference was not statistically significant and was based on very small numbers of cases. Because of small numbers of possible undiagnosed asthma, we excluded the variable as an outcome variable for further analysis. Children born in the US were more likely to report having a family member who had asthma (16.8% vs. 3.2%; p = 0.007). Allergies were more common in the US born children, but the difference was modest and not statistically significant (29.2% vs. 20.3%; p = 0.18).

Self-reported smoking in the home was comparable between foreign and native-born respondents, and there was little maternal smoking during pregnancy in either group. There were trends toward more pests in homes of US born children and more frequently living near heavy motor vehicle traffic in foreign-born children, but these differences were not statistically significant (Table 1).

Place of origin prior to immigration was also collected for immigrants. The majority, 57.1%, came from urban locations in China, 23.8% came from rural locations in China. The remainder came from Taiwan, Hong Kong or other places in Asia. Of all the respondents who immigrated to the US, only 6 were diagnosed with asthma, prohibiting analysis of point of origin or years spent in the US after immigration.

### All age groups

Table 2 presents the results of risk factor tabular analysis for adults and children. We first analyzed all children aged 4–18 together. In subsequent analyses, we divided children into two groups, 4–10 and 11–18 to examine whether risk factors for asthma differed between older and younger children. All variables in Table 2 were also tested in logistic regression models with diagnosed asthma as the outcome variable (data not shown) and only those that remained statistically significant were considered in development of final models. We first analyzed all children aged 4–18 together due to the relatively small overall sample size.

**Table 2:** Risk factors for asthma diagnosis

**Risk factor (yes versus no except where indicated)**	**OR (All subjects)**	**p**	**OR (11–18)**	**p**	**OR (4–10)**	**p**
Site (NEMC versus SC)	1.07	0.85	1.21	0.68	1.53	0.51
Language (Chinese versus English)	2.00	0.036	1.33	0.65	1.60	0.51
Gender (male versus female)	1.15	0.64	0.94	0.89	1.47	0.43
Wheezing	9.00	< 0.001	13.57	0.005	6.00	0.025
Allergies	1.33	0.43	2.71	0.034	0.61	0.43
Family history of asthma	6.40	< 0.001	10.52	< 0.001	4.52	0.07
Born in the US	4.86	< 0.001	3.22	0.038	8.00	0.003
Smoking in the home	1.23	0.53	0.83	0.68	2.15	0.11
Pests seen in or around home	1.17	0.63	1.37	0.50	1.43	0.49
Heavy motor vehicle traffic near home	1.68	0.12	1.61	0.33	1.25	0.64
Family SES (lower versus higher)	2.50	0.22	3.15	0.26	1.80	0.61

Language and reporting living near heavy traffic were strongly and significantly correlated with each other (p < 0.001 using a Pearson correlation matrix) other so we developed two models, one with each variable (Table 3a and Table 3b). In the first model, children born in the US had an OR of being diagnosed with asthma of 4.3 (p = 0.003). Motor traffic was also a significant predictor in this model with an estimated OR of 2.6 (p = 0.012). Finally, a family history of asthma is a very significant predictor with an estimated OR of 6.4 (p < 0.001). In the second model being born in the US and family history of asthma remained highly statistically significant with ORs similar to the first model. In this second model, language was a significant predictor of an asthma diagnosis with an OR of 3.2 (p = 0.003). The psuedo-R^2 ^statistics were very similar (0.226 for the first model versus 0.247 for the second). These statistics suggest that the model with exposure to heavy motor vehicle traffic explained 22.6% of the variance of asthma diagnosis in this population while the model with language explained 24.7%.

**Table 3:** Logistic Regression of children 4–18 years of age

a) Retaining traffic					
**Variable**	**B**	**SE of B**	**Wald**	**p-value**	**Odds Ratio**

BORNUSA	1.5	0.50	9.0	0.003	4.3
MOTOR TRAFFIC	0.9	0.40	6.3	0.012	2.6
FAMILY HISTORY OF ASTHMA	1.9	0.50	13.8	< 0.001	6.4

					
b) Retaining language					

**Variable**	**B**	**SE of B**	**Wald**	**p-value**	**Odds Ratio**

LANGUAGE	1.2	0.39	9.1	.003	3.2
BORNUSA	1.5	0.49	9.4	.002	4.4
FAMILY HISTORY OF ASTHMA	2.0	0.51	14.8	< 0.001	7.2

In no analysis were pests or smoking of any kind significant predictors of an asthma diagnosis. Sex and SES were also not associated with an asthma diagnosis in these data. A history of allergies was not a significant predictor of an asthma diagnosis in the logistic model and it was not significantly associated with asthma diagnosis in the tabular analysis.

### Younger children (4–10 years old)

When divided into two groups (4 to < 11 and 11–18), results were different and the analysis was adversely affected by small sample size, especially in some cells. For the younger children, a logistic regression model that included all variables of interest from Table 2 showed no significant predictor variables of asthma except being born in the US. A family history of asthma no longer has significant predictive value, although the estimated odds ratio is only marginally smaller than the one above (4.5 versus 6.4). The change in significance is most likely due to reduced sample size. The best overall model for this younger group uses just the born in the US variable (OR = 8.0; p = 0.008).

### Older children (11–18 years old)

For the older children, being born in the US loses some of its predictive power. In a univariate model, born in the US remains a significant predictor of asthma (OR = 3.22; p = 0.046). However, a larger model, like the one for younger children, was limited by the small sample size and no variable, including being born in the US, remained significant except for a family history of asthma. In a model including only being born in the US and family history of asthma, the family history variable remained statistically significant (OR = 8.9; p < 0.001) but the born in the US variable is not statistically significant (OR = 2.2; p = 0.22). Removing born in the US from the model marginally increases the association with family history of asthma (OR = 10.4; p < 0.001). In addition to small sample size, there is also significant correlation between born in the US and family history of asthma in this group (p = 0.020 from a Pearson correlation matrix).

Thus, in younger children, being born in the US is the main predictor of asthma diagnosis while in older children having a family member with asthma is the main predictor of asthma in the children included in our survey.

## Discussion

We found a markedly higher prevalence of asthma for children born in the US as compared to those who were foreign-born in an Asian population, enriched with recent Chinese immigrants, as evidenced by the fraction who preferred Cantonese to English. These results confirm findings in a similar Asian population from the same community [22]. The results presented build on the earlier finding by including questions about key environmental and demographic factors that allowed us to test for the possibility that native and foreign born might be surrogates for SES, exposure to poor housing conditions, traffic-related pollution, or tobacco smoke. To the extent that we were able to assess these factors, which were self reported by respondents, they did not explain the observed strong association between asthma diagnosis and place of birth, although heavy traffic remained a significant association in the model with children ages 4–18, consistent with recent studies showing an association between living near highways in the first two years of life and risk of developing asthma [25]. Our findings suggest that factors associated with very early life experiences are significant determinants of risk of developing asthma.

We found a different picture for children 4–10 and children 11–18. The effect of being born in the US dominated in the younger children while the effect of family history of asthma dominated in the older children. We do not know details of the immigration history of the children in the analysis, but the percentage of foreign and native-born children was very similar in both age groups (Table 1). The result is not consistent with the assumption that the same population of younger children grows up to become the older child population in the clinic from which we drew our sample.

One possible explanation for the difference in the age groups, consistent with what we know about the flow of Chinese immigrants into Boston and Chinatown, is that the younger and older populations are distinct. Families that immigrated recently with either younger or older children may move on from using Chinese language health care services after a few years causing turnover in the population. If this were the case the older children could have spent many more years in China after birth than the younger children. Another possibility is that older children, reporting for themselves, gave differential responses compared to parents answering for their younger children.

Other, more standard risk factors were not significant in the age sub-groups, but small numbers limited statistical power. Therefore, we can not really state that, for example, heavy motor traffic (both age groups) or family history of asthma (in children < 11 years old) are not predictors of asthma in the two age categories. The estimated odds ratios for these factors were relatively high (not shown) and in the right direction for these factors, but significance is not achieved because of sample size. In the model with all children (4–18 years old), heavy motor traffic was significant. This is because it is getting close to significant in both age groups so that, when combined, the sample size is large enough to show the effect.

The hygiene hypothesis is a leading candidate to explain early childhood development of susceptibility or resistance to becoming asthmatic. It asserts that several factors have contributed to change the infectious environment during childhood [18]. First proposed in 1986, this theory maintains that Th1 cells are responsible for cell-mediated immunity and that Th1 and Th2 cross-regulate each other. It predicts that allergic disease develops when there are too many Th2 cells and not enough Th1 cells. Two factors studied with respect to the hygiene hypothesis that seem relevant to our findings are hepatitis A and helminthes. Exposure to both is associated with protection against developing asthma and both are more prevalent in developing countries [26].

Depending on age and frequency of exposure, these experiences could plausibly explain the lower incidence of asthma observed in foreign born children in our sample population. Alternative explanations exist, including the possibility that families with asthmatic children are less likely to immigrate to the US, although this seems unlikely to us given the broad consensus that asthma is less prevalent in China than the US.

The data analyzed here were self-reported. In the case of asthma diagnosis, self-report is widely used and our wording was taken from a validated survey instrument that we found performed well in our sample in terms of internal consistency. Nevertheless, we did not fully validate the asthma questions in Chinese or with Chinese immigrant respondents. We are not aware of any Cantonese questionnaire for asthma that is fully validated and that addresses language issues that we previously identified [22]. We were unable to test associations of asthma with place of origin or years lived in the US. Questions about environmental factors are less reliable than direct observation or measurement. Respondents were recruited from the waiting rooms of two urban pediatric clinics that serve large numbers of recent Chinese immigrants. The sample we used can be considered to have over-sampled for children with asthma and children from recent Chinese immigrant families because they are more likely to be present at the clinics. Therefore, our raw prevalence values should not be considered representative of either the US population or the Chinese population in the US.

## Conclusion

We have confirmed that place of birth is a strong predictor of asthma in a Chinese pediatric population. Our findings are consistent with the hygiene hypothesis in that early childhood events would be necessary to account for the association with place of birth and in that several environmental factors including smoking, pests, heavy traffic and SES were not associated with place of birth or, with the exception of traffic, with asthma.

## Abbreviations

SES = Socio economic status

HIPPA = Health insurance portability and accountability act

OR = Odds ratio

Th1 = T lymphocytes associated a proinflammatory response.

Th2 = T lymphocytes associated with atopy and with anti-inflammatory responses.

## Competing interests

The author(s) declare that they have no competing interests.

## Authors' contributions

Doug Brugge oversaw the study design and IRB submission, supervised data collection and took the lead on writing the paper. Angela C Lee did the bulk of the data collection and data management and commented on the analysis and paper. Mark Woodin conducted the statistical analysis and commented on the paper. Christine Rioux did the literature review, wrote sections of the introduction and discussion and commented on the analysis and paper.
